# The Education of Physicians and Dentists

**Published:** 1891-05

**Authors:** H. C. Wood


ARTICLE V.

### THE EDUCATION OF PHYSICIANS AND DENTISTS.

BY PROF. H. C. WOOD.

The American medical professions has in it a multitude of rightly educated physicians, but it comprises also an enormous number who are but partially educated in their profession. Of all places in the universe, in America there are doctors and doctors.

The American medical profession cannot be in any degree held responsible for its condition, not merely because it has no power over its own members after they have entered, but especially because it has no control over the gates through which men flock into it. In some States the law allows any one to set up as a doctor who wishes; while where there is any law regulating the mode of entrance into the profession, such law usually puts the power of granting the right to practice into the hands of the medical college. To be sure the medical college is nominally required to examine the candidates, and to shut out the unfit. Almost any small group of physicians can, however, constitute themselves a medical school, and conduct their examinations so privately that no outsiders can know whether these trials be substance or shadow. The national vice, the imperative desire to get on in the immediate present, fills the land with persons who wish to get the right to practice medicine at the lowest outlay of money, time and labor. For these candidates the schools bid, one against the other; and so the standard falls lower and lower, medical education becomes a farce, and the doors of entrance to practice stand wide open to any one who can raise a few hundred dollars.

At the recent examination for the Army Board, of thirty doctors who had been picked out from among the best graduates, and had been especially prepared for the army examination, only two reached the required standard. I believe myself that not twenty per cent. of the graduates of medicine in America could pass the State examination required in Germany for license to practice. Humiliating though it be, yet it is true that an American medical diploma has in itself no meaning, and that it will never be a true certificate of technical knowledge and education until it is supplemented by the law.

If such language can be appropriately used with reference to the medical profession, medical colleges, medical examinations, and medical diplomas, is it not worth while for all concerned—practitioners, professors, students—to inquire how far it is applicable to existing facts in similar dental relations. The national vice, the imperative desire to get on in the immediate present, it is not unreasonable to suppose, is as potent in dentistry as in medicine, and unless sedulously guarded against, the sequence will correspond, the standard will fall lower and lower and dental education become a farce.
—*Items of Interest.*

